# Rational design of NiFe alloys for efficient electrochemical hydrogen evolution reaction: effects of Ni/Fe molar ratios

**DOI:** 10.1039/d2ra05922c

**Published:** 2022-10-12

**Authors:** Yazid Messaoudi, Hamza Belhadj, Mohamed R. Khelladi, Amor Azizi

**Affiliations:** Laboratoire de Chimie, Ingénierie Moléculaire et Nanostructures, Université Ferhat Abbas Sétif 1 Sétif 19000 Algeria; Unit of Research in Nanosciences and Nanotechnologies (URNN), Center for Development of Advanced Technologies (CDTA), Université Ferhat Abbas Sétif 1 Sétif 19000 Algeria hbelhadj@cdta.dz belhadjhamza@gmail.com

## Abstract

Developing and designing high-performance and stable NiFe electrodes for efficient hydrogen production are the greatest challenges in electrochemical water splitting. In this work, NiFe alloys with different Ni and Fe contents are prepared by a simple electrodeposition method using different molar ratios of Ni/Fe precursors (Ni/Fe; 1 : 3, 1 : 1 and 3 : 1). The obtained NiFe electrode with a molar ratio of 3 : 1 exhibited better electrocatalytic activity for the HER than the other NiFe electrodes with 1 : 3 and 1 : 1 molar ratios. The NiFe (Ni/Fe, 3 : 1) electrode required an overpotential of 133 mV to reach a current density of 10 mA cm^−2^, which was much lower than those of NiFe with molar ratio of 1 : 3 (220 mV), and 1 : 1 (365 mV), respectively. Tafel slope analyses demonstrated that the HER mechanism of NiFe alloy coatings followed the Volmer reaction type. The superior electrocatalytic performance of the NiFe alloy for HER depending on precursor molar ratio of Ni/Fe was attributed to their composition in terms of Ni and Fe content, structure and surface morphology. Specifically, the electrodeposition of the NiFe alloy was obtained in a molar ratio Ni/Fe, 3 : 1, and induced the formation of NiFe layered double hydroxide (LDH) with a nanosheet-array structure. The high electrocatalytic activity of NiFe LDH (Ni/Fe, 3 : 1) confirmed the critical influence of Ni and Fe contents in the alloy resulting in an increase the active surface on the surfaces, which is most likely explained by the higher surface roughness and the low crystallinity structure of NiFe nanosheet-array, supported by ECSA measurement, XRD, SEM and AFM analyses. The present strategy may open an avenue for developing cost-effective, stable and high-performance electrocatalysts as advanced electrodes for large-scale water splitting.

## Abstract 2

Developing and designing high-performance and stable NiFe electrodes for efficient hydrogen production in alkaline medium.

## Graphical abstract

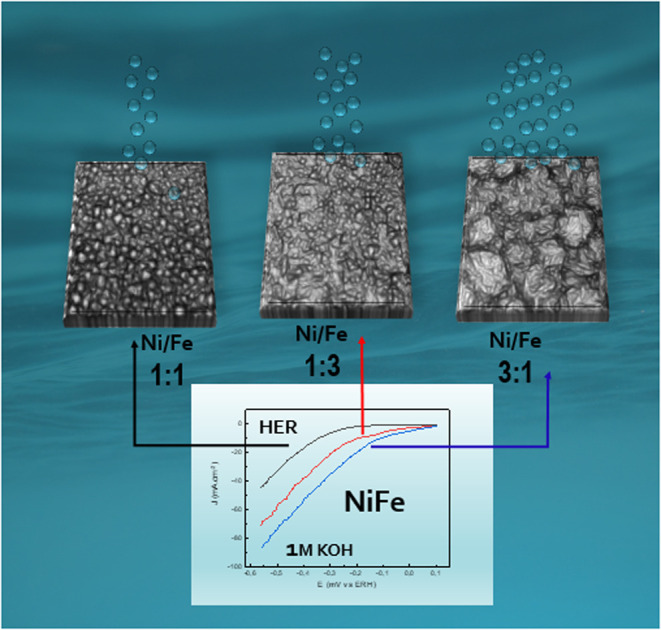

## Introduction

Electrocatalytic water splitting has been widely regarded as one of the most promising approaches for generation of clean hydrogen, which is considered to be an effective alternative to fossil fuels for the future energy sustainability.^1,2^ At present, the highest efficiency electrocatalysts for water splitting still belong to the commercial noble metal based materials such as Pt for the hydrogen evolution reaction (HER) and Ir/Ru oxide materials for the oxygen evolution reaction (OER).^1,3^ However, the high cost and scarcity of these precious metals largely hinder their large-scale application.^4^ Therefore, tremendous research efforts have been focused on the investigation of non-noble metal alternatives such as nickel alloys,^5,6^ transition metal nitride,^7^ sulfides^8^ and metal oxides.^9,10^ Among various alternatives, nickel-based binary alloys with iron metal (NiFe) have attracted considerable research attention during the last decades because of their numerous interesting properties.^11,12^ In particular, NiFe layered double hydroxide (NiFe LDH) has been intensively investigated as electrocatalysts for water splitting due to their high catalytic activity, characteristic layered structure, and easily scale preparation.^13,14^ The NiFe alloy and NiFe LDH electrodes have been prepared by various methods, such as magnetron sputtering,^15^ hydrothermal treatment,^16^ mechanical alloying^17^ and electrodeposition technique.^18,19^ Among them, electrodeposition is considered as an easy and effective technique for preparation of alloy coating due to genuine reason of its low cost and greater flexibility to tailor the properties, like composition, phase structures and morphology of the coatings.^20,21^ The electrodeposition of NiFe alloys has been studied by several researchers, and it has been shown that NiFe alloy coating can be varied by controlling the deposition parameters such as applied potential, ion concentration, pH and bath temperature.^22,23^ Luo *et al.*, developed a simple and versatile electrodeposition method to fabricate high-efficiency catalysts of NiFe nanosheets film in an equimolar metal salt precursor.^24^ In another study, Kardaş *et al.*, revealed that the presence of nickel with iron increases the electrocatalytic activity of the coating for the HER in 1 M KOH solution when compared to nickel and iron coatings individually, and that the HER activity of the coatings depends on the chemical composition of the composite coatings.^25^ Additionally, it is well known that the catalytic performances of electrode depended strongly on the mainstream characteristics of NiFe electrode such as a large active surface area, electrochemical stability, good electrical conductivity, low overpotential and selectivity.^25,26^ By changing the composition of the electrolyte and simple craft adjustment, electrodeposition has the ability to get the optimized catalyst design with desired nanoarchitecture and composition, which cater to the trend of simple strategies to develop new electrode for water splitting.^27^ Tedim *et al.* investigated the effect of Ni/Fe ratio and crystallinity on Ni–Fe LDH formation and OER activity.^28^ From the results of this investigation, no evidence was found for any impact of the Ni/Fe ratio in the efficiency of the OER. However, the impact of the specificity of the atomic arrangement in the LDH and of its composition on the catalytic efficiency of the material are still unknown. There are tremendous efforts to develop this strategy to improve the performance of NiFe catalysts including properly choosing the optimal ratio, controlling the size/morphology and understanding the catalytic mechanism, however, the recognition of active sites and the mechanism of this NiFe catalyst for OER and HER are still controversial. Furthermore, the existence of metal alloy NiFe/NiFe LDH and the synergistic effect of Ni and Fe which is very important has not been fully investigated for HER and therefore, it would be important to look into their role in enhancing the HER activities. To highlight the effect of precursor molar ratio of Ni/Fe and deposition surface composition, we herein report an effective and facile strategy to prepare nickel–iron (NiFe) alloy with enhanced surface area and intrinsically high catalytic activity by controlling the growth of Ni and Fe deposit. By rationally designing the composition, morphology, and structural characteristics, the nickel iron alloy were fabricated in a scalable and low-cost manner by electrodeposition method. The impacts of different Ni/Fe molar ratios (Ni : Fe; 1 : 3, 1 : 1, 3 : 1) on the kinetics of NiFe deposited on copper substrates and the electrocatalytic activity of the developed NiFe for HER were investigated. Electrochemical measurement of NiFe electrode for HER in alkaline electrolyte were studied and the structural, compositional and morphological changes induced by Ni and Fe incorporation were compared in term of electrocatalytic activity toward HER. Based on the experimental results, we found that the catalytic rate of HER catalyzed by NiFe with molar ratio of 3 : 1 exhibits excellent HER performance with a low potential of about 133 mV *vs.* RHE at current density of 10 mA cm^−2^, a low Tafel slope of 179.19 mV dec^−1^ in 1 M KOH. The remarkable electrocatalytic performance of NiFe could be attributed to the unique nanosheet-array structures of NiFe LDH, with the large active surface area ECSA (1140.75 cm^2^) and the surface roughness of NiFe (223.72 nm), leading to the improved charge transfer kinetics and intrinsic activity of the NiFe LDH catalyst. These results were further correlated to the NiFe alloys properties. This therefore provides insights into the structure–property effect on the catalytic activities for the HER.

## Experimental

### Materials and reagents

Nickel(ii) sulfate hexahydrate (NiSO_4_·6H_2_O) and iron(ii) sulfate heptahydrate (FeSO_4_·7H_2_O) were obtained from Biochem Chemopharma (Canada). Sodium hydroxide (NaOH) and potassium hydroxide (KOH) were purchased from VWR. Nitric acid (HNO_3_, 69%) from Sigma-Aldrich. Double distilled water was used as a solvent. All the materials were reagent grade and used without further purification.

### Electrodeposition of NiFe electrode

Before the electrodeposition experiments, the copper substrates (0.5 cm × 0.5 cm) were mechanically wet polished with emery paper, then cleaned ultrasonically in acetone and ethanol for 15 minutes. Afterward, the copper substrates were washed in distilled water and activated in 0.5 M HNO_3_ for 30 second at room temperature. NiFe electrodes were electrochemically deposited onto the surface of copper sheet using different molar ratios (Ni : Fe; 1 : 3, 1 : 1, 3 : 1) of NiSO_4_·6H_2_O and FeSO_4_·7H_2_O. The electrodeposition of NiFe thin films were performed on a electrochemical workstation in a three-electrode cell using the cleaned Cu substrate as the working electrode, a saturated Ag/AgCl as the reference electrode and a platinum (Pt) as the counter electrode. The electrolyte (100 mL) contained both Ni and Fe precursors by mixing appropriate amounts with equimolar ratio of NiSO_4_·6H_2_O (0.15 M) and FeSO_4_·7H_2_O (0.15 M) in deionized water. Three molar ratios of Ni *versus* Fe precursors were investigated (Ni/Fe; 1 : 3, 1 : 1 and 3 : 1).The electrodeposition of NiFe with different molar ratios were performed at an applied potential of −1.0 V *vs.* Ag/AgCl, for 10 min at room temperature, under an inert atmosphere. Finally, the obtained NiFe electrodes were washed with deionized water and dried in air.

### Material characterization

The as-prepared NiFe alloy catalysts were examined by X-ray diffraction (XRD) on a Bruker D8 Discover X-ray Diffractometer with a CuKα beam radiation (40 kV, 40 mA, *λ* = 1.5406 Å). The composition and morphology were studied by field emission scanning electron microscopy JEOL 7001F with energy-dispersive X-ray spectroscopy (EDX) at an operating voltage of 15 kV and 5 kV, respectively. The surface morphology, thickness and roughness were examined by recording Atomic Force Microscopy (AFM) images with an MFP-3D AFM (Oxford Instruments Asylum Research) in contact mode with Si cantilever. Values of Root Mean Square (RMS) roughness were calculated from the height values in the atomic force microscopy images using the commercial AR software. These measurements were performed using the peak force tapping mode on fresh prepared samples.

### Electrochemical measurements

Electrochemical measurements of NiFe electrode were conducted in a classical three-electrode configuration cell containing 1 M KOH aqueous solution as the electrolyte using an electrochemical workstation, potentiostat/galvanostat (Bio-Logic SP-300). The developed NiFe alloy coatings were used as the working electrode (with a constant geometrical surface of 0.25 cm^2^). The counter electrode was a large Pt foil, while Ag/AgCl electrode was used as a reference electrode. All potential data given in this study were converted and referred to the reversible hydrogen electrode (RHE) scale by the following formula:^29^

1 *E*_RHE_ = *E*_Ag/AgCl_ + 0.059 × pH + 0.197 V

HER performance was achieved by linear sweep voltammetry (LSV) and Tafel measurements in 1 M KOH. The electrochemical impedance spectroscopy (EIS) curves were obtained at frequencies of 100 kHz to 0.1 Hz. All electrochemical characterisation were carried out in alkaline electrolyte (1 M KOH), which was purged with N_2_ for 15 min prior to the measurements to remove dissolved oxygen.

## Results and discussion

### Surface morphology and structural properties of NiFe coating

**Fig. 1:**
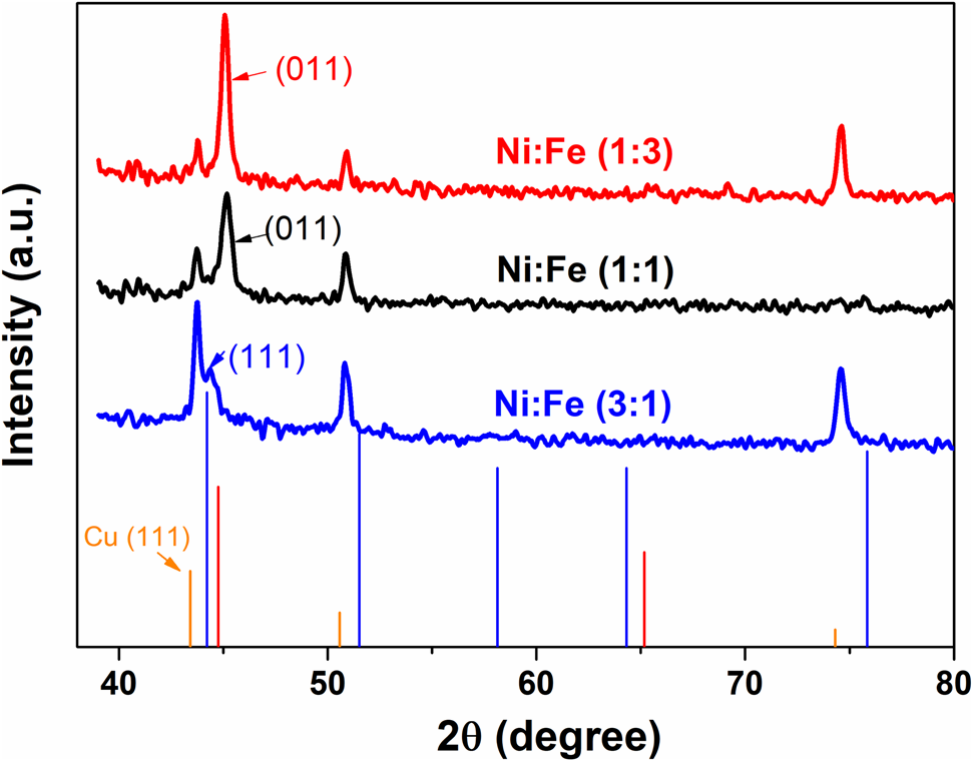
X-ray diffraction patterns of NiFe alloys deposited at various molar concentration ratios (Ni/Fe; 1 : 3, 1 : 1 and 3 : 1).

**Fig. 2:**
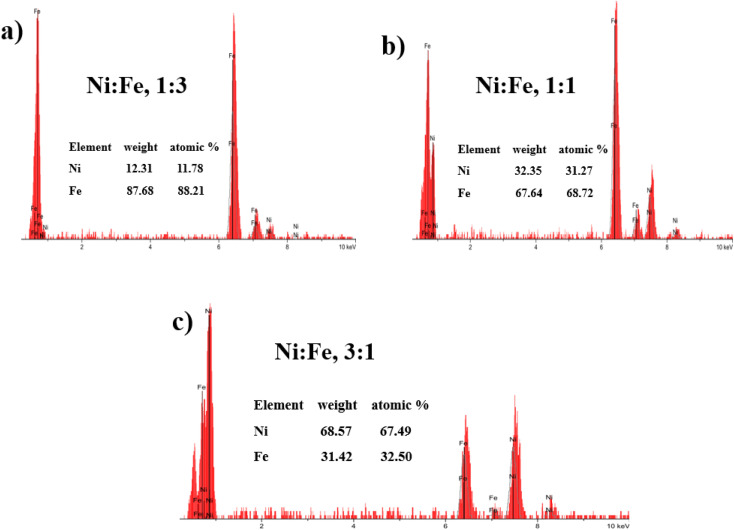
Energy Dispersive X-ray analysis (EDX) analysis of electrodeposited NiFe alloys: (a) Ni : Fe, 1 : 3, (b) Ni : Fe, 1 : 1, (c) Ni : Fe, 3 : 1.

**Fig. 3:**
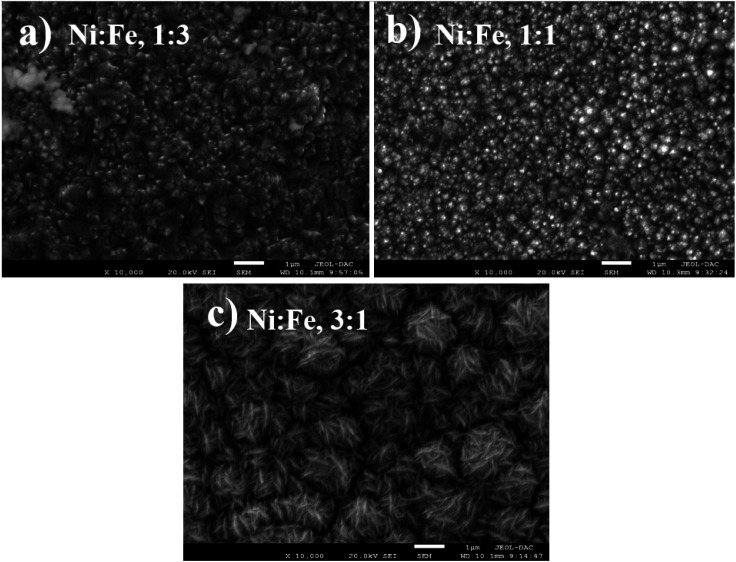
SEM images of NiFe alloys deposited at various molar concentration ratios: (a) Ni : Fe, 1 : 3, (b) Ni : Fe, 1 : 1, (c) Ni : Fe, 3 : 1.

**Fig. 4:**
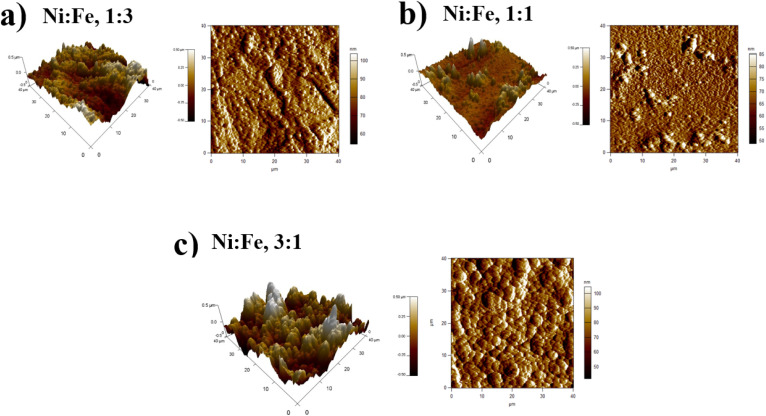
2D and 3D-AFM images of Ni–Fe alloy coatings deposited at various molar concentration ratios (a) Ni : Fe, 1 : 3, (b) Ni : Fe, 1 : 1 and (c) Ni : Fe, 3 : 1.

X-ray diffraction (XRD) was used for the structural characterization of the NiFe alloy coating. Here, we analyzed the X-ray spectra of as-prepared NiFe electrocatalysts obtained by electrodeposition technique in different Ni/Fe electrolyte ratio. The resulting diffraction patterns are shown in Fig. 1. XRD analysis represents the diffraction pattern of Cu substrate in which the peaks at 2*θ* value of 43.28°, 50.40° and 74.81° correspond to (111), (200) and (220) planes respectively (JCPDS 89-2838).^30^ Besides, a strong peak from NiFe coating in different molar ratios (Ni/Fe) are identified at 43.5°. The range of variation of the diffraction angle was 43.5° < 2*θ* < 45.5°. The NiFe coating with a higher content of iron (Fe) shows a broad peak corresponding, in increasing order of 2*θ*, to the (011) reflections of the f.c.c, which is in good agreement with the standard JCPDS data (37-0474) for the NiFe and having cubic crystal structure.^31^ It was clearly seen that, at the molar ratio of Ni/Fe, 1 : 3, the reflection peaks became sharper indicating the enhancement of crystallinity.^32^ The crystallite size of NiFe were calculated by Debye–Scherrer formula,^33^ which shows similar trend of increasing crystallite size with increase in iron concentration. The average crystallite size of NiFe samples was 54.86, 114.60 and 168.45 nm for the NiFe deposited at different molar ratios of Ni/Fe precursors: (3 : 1), (1 : 1) and (1 : 3) respectively. The chemical composition of the electrodeposited NiFe films was measured using Energy-Dispersive X-ray spectroscopy (EDX) with the result shown in Fig. 2. Inset of Fig. 2, presents the EDX quantitative analyses of NiFe both in weight (wt%) and atomic percentages (at%). According to the Fig. 2, it can be found that in all samples, coating composition of NiFe alloys obtained from electrolyte depend significantly on the concentration molar ratios of Ni and Fe precursors. Quantities of incorporated Fe in alloys from electrolyte (Ni/Fe, 1 : 3) increased during time of electrodeposition. The same dependence is observed with respect to deposited Ni from electrolyte (Ni/Fe, 3 : 1). However, at equimolar concentration ratio (Ni/Fe, 1 : 1), the amount of iron in the NiFe alloy increased substantially, which favors its deposition. In order to examine the effect of precursor concentration ratio on the morphology of NiFe deposit, a scanning electron microscopy (SEM) technique was used. The surface morphology of NiFe alloy coatings, deposited in different concentration of Ni and Fe precursors are shown in Fig. 3. The SEM images show a significant variation in surface morphology, indicating that the change in a molar ratio of Ni/Fe precursor would affect the chemical composition of the coating films as shown previously in EDX data. It is clear from Fig. 3a that the surface morphology of NiFe coatings deposited at low concentrations of Ni, (Ni/Fe, 1 : 3) presents in the form of micro–nanocones structure which were similar to those reported in the literature.^34,35^ At an equimolar concentration ratio, it can be observed that the surface morphology of NiFe (1 : 1) coatings is found to be agglomerate that is distributed homogenously over the copper substrate. In contrast, at high Ni concentrations (Ni/Fe, 3 : 1), the formation of vertically aligned nanosheet array structures are observed. The nanosheet-array structures of NiFe coatings can most likely be assigned to a NiFe layered double hydroxides (NiFe-LDH) and the shape of this morphology were similar to those NiFe electrocatalyst reported by Wu *et al.*^36^ and Tang *et al.*^37^ Additionally, it was found that the nanosheet-array structures of LDH sheets were formed once the concentration of Ni rose to a very high concentration. Briefly, the mechanism for the formation of these layered double hydroxides, is due to the partial replacement of Fe^3+^ by Ni^2+^ ions resulting Ni(OH)_2_ which consists of a monolayer of nanosheets.^38^ The self-assembly of NiFe double hydroxide layers were directly deposited on Cu substrate and generated nanosheet array.^36,39^ For more investigation, the atomic force microscopy (AFM) is used to investigate the surface roughness and morphology of the electrodeposited NiFe electrodes. Accordingly, a three-dimensional AFM image of the NiFe alloy coatings deposited at different molar ratio have been recorded, and are shown in Fig. 4. Furthermore, the average surface roughness for each sample was calculated for a scan area of 40 × 40 μm^2^ and expressed as the root mean square (rms). The average surface roughness of NiFe with different molar concentration ratios are 167.51 nm, 85.12 nm, and 223.72 nm for Ni/Fe; 1 : 3, 1 : 1 and 3 : 1 respectively. It can be clearly seen from AFM images that, the morphology and the surface roughness of the NiFe are different. The analysis of AFM images revealed that the NiFe thin films prepared with Ni/Fe molar ratio of 1 : 1 exhibit the growth of small grains distributed across the surface of the substrate (Fig. 4b). As can be clearly seen that the surface roughness values of catalysts depends strongly on the composition ratio of the NiFe alloys (Fig. 4), due to the formation of different nanostructure morphology (Fig. 3). Interestingly, the electrodeposition of NiFe alloy with 1 : 1 and 3 : 1 molar concentration ratio causes an increasing of surface roughness from 85.12 to 223.72 nm, respectively. This difference in roughness values confirms that the high ratio of Ni content has influenced the nucleation and growth mechanism from the bottom to the surface of NiFe resulting in layered double hydroxides nanosheet-array formation, which is confirmed by SEM (Fig. 3c).

### Electrochemical characterization

**Fig. 5:**
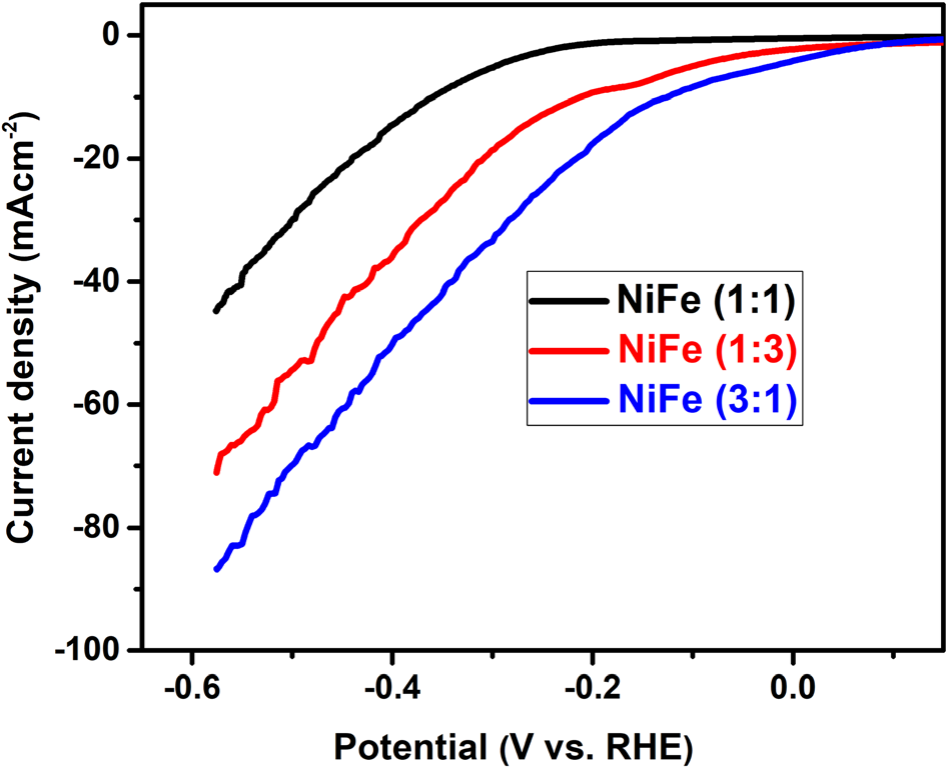
Linear sweep voltammetry curves of NiFe electrode with different Ni/Fe molar ratios in 1 M KOH, sweep rate: 5 mV s^−1^.

**Fig. 6:**
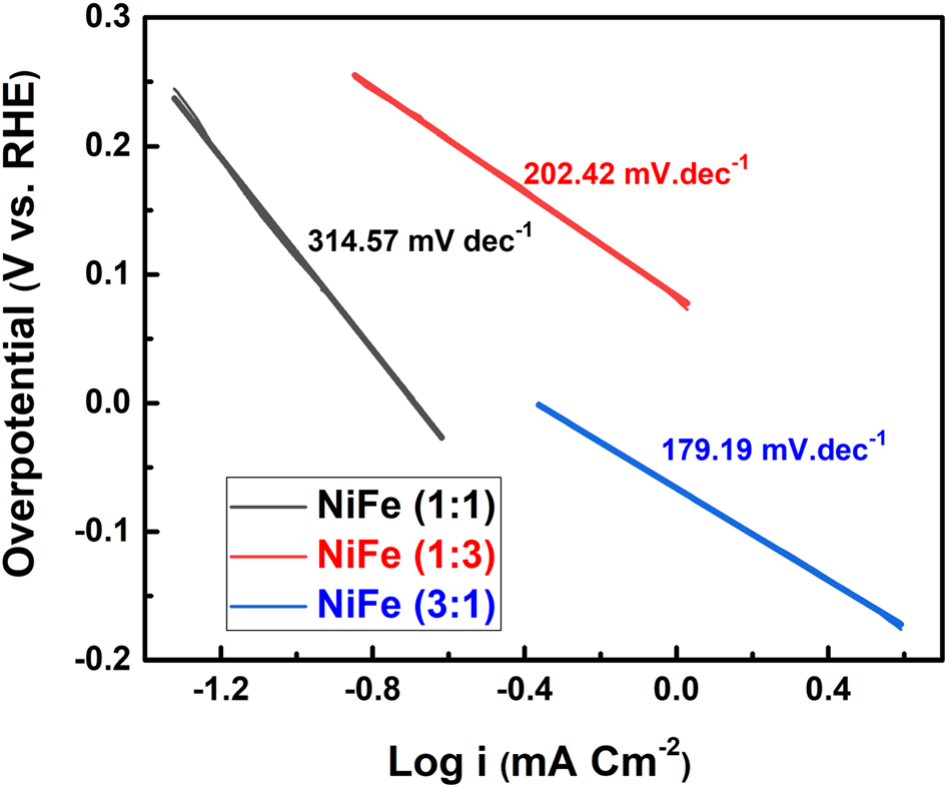
Nyquist plots for the NiFe electrocatalysts with different Ni/Fe molar ratios.

**Fig. 7:**
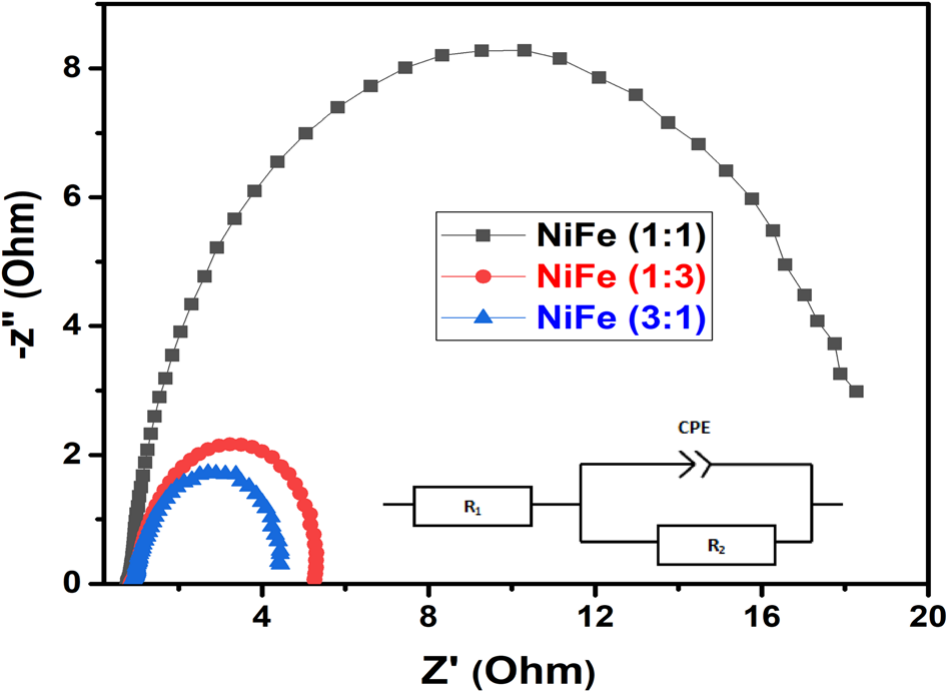
The corresponding Tafel plots of different NiFe electrodes for the HER.

In order to evaluate the electrocatalytic activity of NiFe electrodes (Ni/Fe; 1 : 3, 1 : 1 and 3 : 1) for hydrogen evolution reaction, the linear sweep voltammetry (LSV) analysis was used in alkaline solution. LSV curves of different samples are demonstrated in Fig. 5, where the data were recorded at a scan rate of 5 mV s^−1^ in 1 M KOH electrolyte. Generally, in the LSV curves, the overpotential required for delivering current densities of 10, 20, 40 mA cm^−2^ can be considered as a criterion of electrocatalytic activity. For comparison, it has been observed that, the overpotentials for the NiFe (Ni/Fe, 3 : 1) are as low as 133, 225, and 342 mV, for the cathodic current densities of 10, 20, and 40 mA cm^−2^, respectively. In contrast, the overpotentials required to drive the cathodic current densities of 10, 20, and 40 mA cm^−2^ are 220, 315, and 424 mV for the NiFe (Ni/Fe, 1 : 3), and 365, 446, and 552 mV for the NiFe (Ni/Fe, 1 : 1) (Fig. 5). The lower onset potential and much higher current density demonstrate that the NiFe (Ni/Fe, 3 : 1) have excellent electrocatalytic activity towards HER than other electrodes (Ni/Fe, 1 : 3 and 1 : 1). Furthermore, the Tafel slopes of NiFe as-prepared samples are investigated to reveal the mechanism and the reaction kinetics. The corresponding Tafel plots calculated according to the LSV polarization curves are shown in Fig. 6. The linear portions of Tafel plots were fitted to the Tafel equation: *η* = *a* + *b* log *j*, where *η* is overpotential, *j* is the current density, and *b* is the Tafel slope.^40^ Evidently, the NiFe (Ni/Fe, 3 : 1) electrode exhibits a small Tafel slope of 179.19 mV dec^−1^, which is much lower than those for the other two NiFe electrodes 1 : 3 (202.42 mV dec^−1^) and 1 : 1 (314.57 mV dec^−1^). The low Tafel slope of the NiFe electrode indicates better hydrogen production kinetics of its surface. Furthermore, it can be clearly observed from Tafel slope analysis that the HER activity of the electrocatalysts is mainly determined by the prior water dissociation process through Volmer reaction pathway.^41,42^ To determine the true surface area and to further investigate the electrochemical activity of the electrode materials, the electrochemical impedance spectroscopy (EIS) measurements were performed in a frequency range between 100 kHz and 0.1 Hz in 1 M KOH (Fig. 7). The Nyquist plots show semicircles for all of the electrocatalysts and were fitted well with the equivalent circuit composed of series resistance (*R*_s_), charge transfer resistance (*R*_ct_), and a constant phase element CPE (inset, Fig. 7). The semicircle observed in the Nyquist plot of NiFe samples are fitted to simulate the interfacial electrochemical double layer (*C*_dl_). The *R*_s_ (*R*_1_) and *R*_ct_ (*R*_2_) and *C*_dl_ values obtained after fitting the Nyquist plots are summarized in Table 1.

**Table 1:** The obtained values of solution resistance (*R*_s_), charge-transfer resistance (*R*_ct_), double layer capacitance (*C*_dl_), and electrochemical active surface area (ECSA)

NiFe samples	*R*_1_: *R*_s_ (Ω)	*R*_2_: *R*_ct_ (Ω)	*C*_dl_ (mF cm^−2^)	ECSA/cm^2^
Ni/Fe 1 : 1	1.014	18.14	11.04	276.00
Ni/Fe 1 : 3	1.038	4.412	25.68	642.00
Ni/Fe 3 : 1	1.055	3.487	45.63	1140.75

**Fig. 8:**
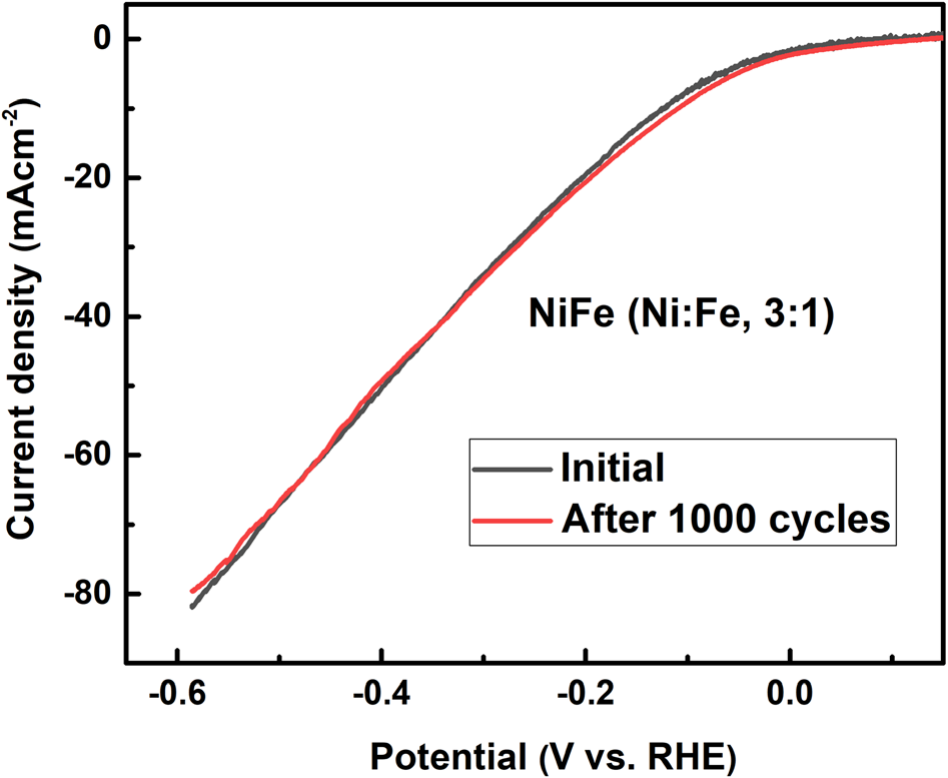
Polarization curves of NiFe with molar ratio of 3 : 1 before and after 1000 cycles.

As can be seen from the results summarized in Table 1, NiFe with 3 : 1 molar ratio has the lowest charge transfer resistance than those of NiFe with molar ratio of 1 : 3 and 1 : 1 respectively, indicating the highest electronic conductivity, fastest charge transfer rate and most facile catalytic kinetics of NiFe (Ni/Fe, 3 : 1) toward HER among all the catalysts. Obviously, in the high frequency region, the low value of solution resistance (*R*_s_) represents uncompensated solution resistances, which were nearly similar for all the NiFe electrodes. Measuring double layer capacitance (*C*_dl_) might be an appropriate approach to probe the electrochemical active surface area (ECSA) of different NiFe electrode. Thus, the ECSAs of all of the NiFe electrodes were calculated from their double-layer capacitance (*C*_dl_) using the equation (ECSA = *C*_dl_/*C*_S_), where *C*_S_ is the specific capacitance and is 0.040 mF cm^−2^ in 1 mol L^−1^ KOH.^43,44^ As shown in Table 1, the ECSA for the NiFe (Ni/Fe, 3 : 1), NiFe (Ni/Fe, 1 : 3) and NiFe (Ni/Fe, 1 : 1) found to be 1140.75, 642.00 and 276.00 cm^2^, respectively. The higher ECSA value of NiFe with a Ni/Fe ratio of 1 : 3 signify better electrochemical activity and excellent HER kinetics compared with other NiFe electrodes due to a superior number of active sites exposed to surface reactions.^29^ It is well known that the number of active surface sites (ECSA) increases with decreasing crystallite size.^45^ The trend of ECSA (Table 1) is in good agreement with the electrocatalytic activity results (Fig. 5), indicating that the HER performance is related to the number of active surface sites capable of interacting with H intermediate species. Generally, the HER efficiency of the NiFe (Ni/Fe, 3 : 1) electrode can be explained by a synergistic combination of the electrocatalytic components and/or by increasing geometric surface area of the electrode. It can be seen from EDX analysis in Fig. 2 and 6 that the Tafel slope value and charge transfer resistance (Table 1) of the NiFe alloy coatings with molar ration of Ni/Fe, 1 : 1 and 1 : 3 was reduced with increasing Fe content of the coatings. Solmaz *et al.*, reported that the HER activity increased with higher iron contents, which can be related to the synergistic interaction between iron and nickel.^25^ Interestingly, although the amount of iron was lower (31.42 wt%), the electrocatalytic performance of NiFe with a Ni/Fe molar ratio of 3 : 1 showed highly efficient electrocatalytic activity toward HER. Thus, the highly improved HER performance of NiFe (Ni/Fe, 3 : 1) is mainly due to small crystallite size (54.86 nm), high average surface roughness (223.72 nm) and large number of exposed active surface sites (1140.75), which were confirmed by XRD, AFM analysis and ECSA measurement. In addition, from SEM images of NiFe with molar ratio of 3 : 1 (Fig. 3c), a typical nanosheet-array structure is observed, which is assigned to the NiFe layered double hydroxide nanosheets.^36^ It was found that the NiFe-LDH (Ni/Fe, 3 : 1) exhibits a good electrocatalytic activity with a small onset overpotential. This result is consistent with previous studies reported in the literature.^46–48^ However, a detailed investigation and identification of the stoichiometry of the crystalline phase of NiFe-LDH require more advanced measurements and will be a subject of further examination. Moreover, the stability of HER catalysed by the NiFe LDH electrode with molar ratio of 3 : 1 was conducted by continuous cyclic voltammetry measurement before and after 1000 cycles in 1 M KOH. As shown in Fig. 8, the LSV curve obtained after 1000 cycles shifts slightly to higher overpotentials indicating the high catalyst stability for HER in the long-term operation. Finally, we believe that the research on NiFe alloy coating with different molar ratios and their electrocatalytic behavior in alkaline electrolyte will facilitate the detailed understanding of NiFe based catalysts for use in electrochemical conversion and energy storage.

## Conclusion

Ni–Fe alloy coatings were electrodeposited in different molar ratios of Ni/Fe precursors (Ni/Fe; 1 : 3, 1 : 1 and 3 : 1) and their electrocatalytic activity towards alkaline water splitting for hydrogen evolution reaction (HER) were investigated. The resultant NiFe catalyst with molar ratio of 3 : 1 exhibit excellent electrocatalytic performances toward HER in 1 M KOH, which requires overpotential of 133 mV for HER to reach 10 mA cm^−2^, which is superior to that of NiFe electrode with molar ratio of 1 : 3 and 1 : 1. As one of the most promising HER catalysts, NiFe layered double hydroxide (LDH) can be rationally tailored *via* tuning the molar ratio of Ni/Fe precursors at 3 : 1 to achieve enhanced electrocatalytic performance. Furthermore, the NiFe LDH nanosheet array electrodes show excellent stability after 1000 cycles without a significant degradation. The improved electrocatalytic performance of NiFe LDH (Ni/Fe, 3 : 1) can be attributed to the synergetic effects of the Ni and Fe sites, the increased surface roughness and high number of active sites, which are confirmed by structural, morphological analysis and electrochemical measurement. The above results elucidate not only the role of the precursor ratio in the electrodeposition process but also give a new insight into materials design for electrocatalysis applications.

## Conflicts of interest

Authors declare no conflicts of interests.

## Supplementary Material
